# A decadal perspective on north water microbial eukaryotes as Arctic Ocean sentinels

**DOI:** 10.1038/s41598-021-87906-4

**Published:** 2021-04-16

**Authors:** Nastasia J. Freyria, Nathalie Joli, Connie Lovejoy

**Affiliations:** 1 Institut de Biologie Intégrative et des Systèmes, Université Laval, Quebec, QC Canada; 2 Département de Biologie, Institut de Biologie Intégrative et des Systèmes, Université Laval, Quebec, QC G1R1V6 Canada; 3 Institut de Biologie de L’École Normale Supérieure (IBENS), 75005 Paris, France

**Keywords:** Microbial communities, Environmental microbiology, Marine biology, Biodiversity, Biogeography, Microbial ecology, Ecology, Microbiology, Ocean sciences

## Abstract

The North Water region, between Greenland and Ellesmere Island, with high populations of marine birds and mammals, is an Arctic icon. Due to climate related changes, seasonal patterns in water column primary production are changing but the implications for the planktonic microbial eukaryote communities that support the ecosystem are unknown. Here we report microbial community phenology in samples collected over 12 years (2005–2018) from July to October and analysed using high throughput 18S rRNA V4 amplicon sequencing. Community composition was tied to seasonality with summer communities more variable than distinct October communities. In summer, sentinel pan-Arctic species, including a diatom in the *Chaetoceros socialis-gelidus* complex and the picochlorophyte *Micromonas polaris* dominated phytoplankton and were summer specialists. In autumn, uncultured undescribed open water dinoflagellates were favored, and their ubiquity suggests they are sentinels of arctic autumn conditions. Despite the input of nutrients into surface waters, autumn chlorophyll concentrations remained low, refuting projected scenarios that longer ice-free seasons are synonymous with high autumn production and a diatom dominated bloom. Overall, the summer sentinel microbial taxa are persisting, and a subset oceanic dinoflagellate should be monitored for possible ecosystem shifts as later autumn ice formation becomes prevalent elsewhere.

## Introduction

Single-celled microbial eukaryotes include a number of pan-Arctic species that are confined to the Arctic, and whose cultured representatives do not tolerate temperatures typical of warmer oceans^1,2^. These sentinel species, which include diatoms, and small flagellates would be predicted to be sensitive to changing Arctic conditions. The diverse Arctic marine microbial communities support higher trophic level across the Arctic^3^, including the North Water region (*Pikialasorsuaq*) in Northern Baffin Bay between Ellesmere Island and Greenland. The region is exceptionally productive with an initial precocious phytoplankton bloom followed by ongoing production due to local geography. At the top of the North Water, a narrow strait favors the formation of an ice bridge and high winds that prevent ice accumulation to the south, creating a recurring polynya. The presence of only thin ice means that light is available for photosynthesis sooner than elsewhere in the Arctic. Once the ice bridge breaches, productivity is sustained over the summer because of the north flowing current along the Greenland coast that brings nutrient rich Atlantic Water onto the shelf. The nitrate rich Atlantic Water is deflected anticlockwise and eventually moves southward following the Baffin Current^4,5^. These nitrate rich waters merge with silica rich Pacific origin water from Nares Strait and Canadian Arctic Archipelago, and the supply of nutrients to the euphotic zone maintains the prolonged phytoplankton productivity in the North Water^6^. The resulting high nutrient, high mixing regime^7^ can favor diatoms that are a valuable food resource for the *Calanus*^8^ zooplankton, which supports the large marine mammal and bird populations. In years when the ice bridge fails to form or breaks out early, thicker ice accumulates along the coasts of Ellesmere and Baffin islands, with effects on local regional productivity, highlighting the East–West differences in the North Water^9,10^.

Surface waters in the North Water are generally ice-free from late April to late October, although this may vary^10^. From the 1990s when the satellite record began, surface chlorophyll tended to be detected first on the Eastern side of the North Water, but in recent years both early open water and early chlorophyll have been detected further north and even into the Kane Basin^10^. However, the picture is incomplete since remote sensing fails to detect chlorophyll below the surface and maximum biomass is frequently found in a subsurface chlorophyll maximum (SCM). SCMs in the Arctic Ocean typically form in stratified water columns where nutrients are depleted in the surface but light is available at or below a nutrient rich pycnocline^11^. SCMs are reported to occur on both sides of the North Water^11,12^. The SCM on the western side forms below fresher Arctic upper waters atop Pacific influenced water that persists in the Canadian Archipelago and Nares Strait^13^. The SCM on the eastern side sits above Atlantic Water flowing north carried by the West Greenland Current.

The impact of climate driven oceanographic processes on primary production patterns and species composition is not well understood^5^ but surmised to be important^14^. At least one model based on remote sensing data suggest longer open water could lead to wind driven mixing, nutrient entrainment and a second autumn phytoplankton bloom^15,16^. Such a scenario would replace current models based on a single bloom followed by surface nutrient drawdown and low chlorophyll production by resident summer Arctic sentinel species for the rest of the ice-free season. However, the species composition and magnitude of any autumnal bloom is poorly understood due to limited sampling later in the season.

The seasonal and spatial variability in light and nutrients has an impact on marine microbial eukaryotes, including phytoplankton species assemblages^17^. The recent increases in multiyear sea-ice melt and glacial melt also affects the stratification, and potentially the micronutrient budget within the North Water region, with consequences for species selection. Joli et al*.*^12^ suggested micronutrients such as iron from glaciers and the Greenland Ice Sheet, would have favored *Pseudo-nitzschia*, which dominated the phytoplankton community at 20 m depth in 2013. Any such phytoplankton species change could have ecological implications for the entire food web^18,19^. Despite the importance of the North Water, in common with most Arctic Seas, there have been few studies focusing on species variability over time and space. In an effort to disentangle season versus interannual and spatial differences, we investigated the variability of marine microbial eukaryotes from two sides of the North Water region. Samples and oceanographic data were collected from the surface and the SCM over the time period of 2005–2018 from July to October. Communities of microbial eukaryotes were identified by way of amplicons targeting the V4 region of 18S rRNA (rRNA) and the 18S rRNA gene (rDNA). We then applied multivariate and comparative statistics to link differences among communities with environmental drivers, to provide a high-level overview on how microbial eukaryotic communities respond to seasonal changes in the context of multi-annual variability. We explored and monitored biodiversity of Arctic phytoplankton community in a decadal perspective to determine specialist taxa and potential for loss of sentinel taxa in a changing Arctic.

## Results

### Environmental conditions in the North Water

**Figure 1:**
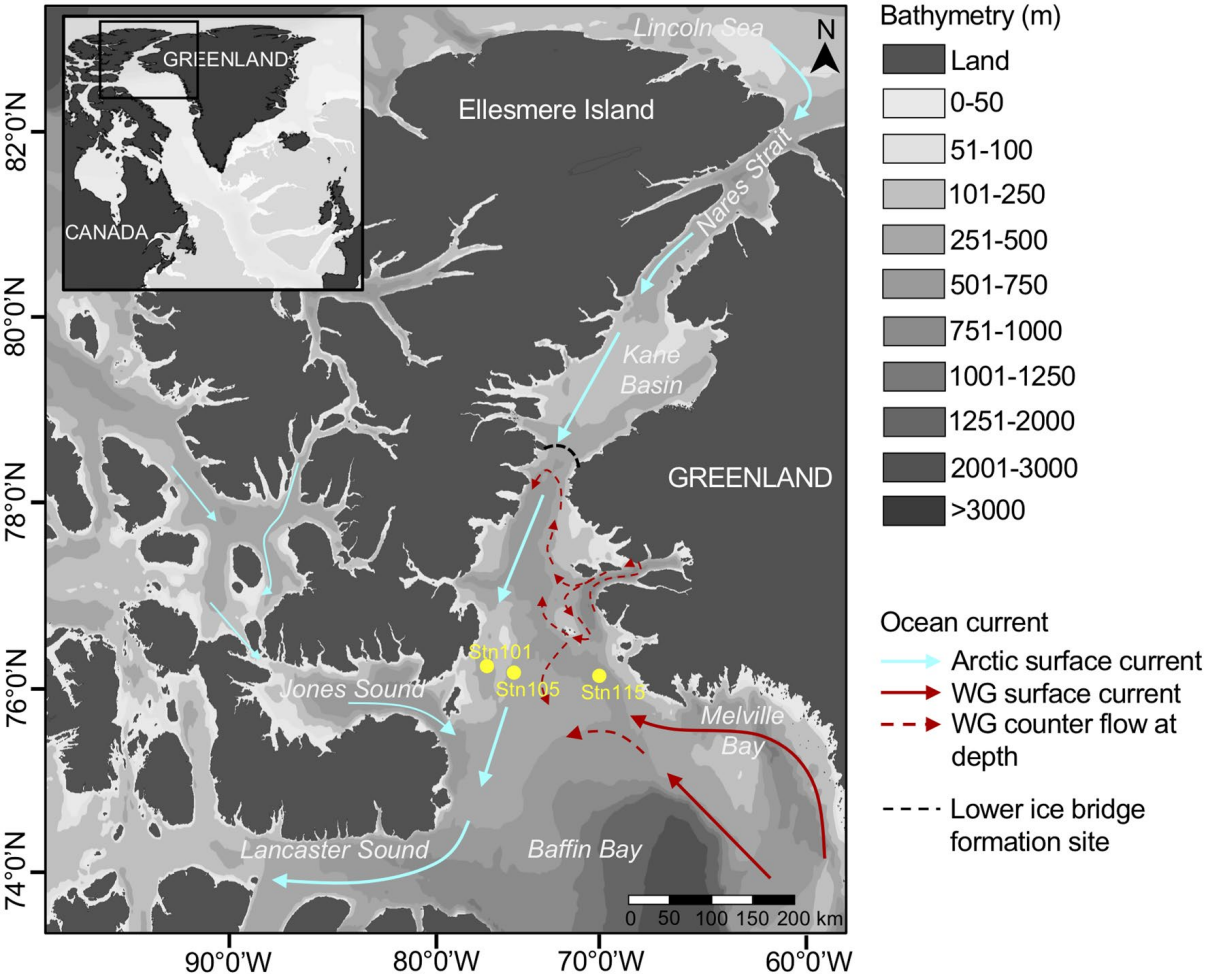
Map of the sampling region. Circulation within the North Water region from Melling et al*.*^62^. Light blue arrows show colder and less salty surface water and red arrows display warmer and saltier surface water. Samples are from the Greenland side (Stn115) and the Canadian side (Stn101 and Stn105). West Greenland current (WG). The map was generated using Ocean Data View (v.4.7.8, http://odv.awi.de).

**Figure 2:**
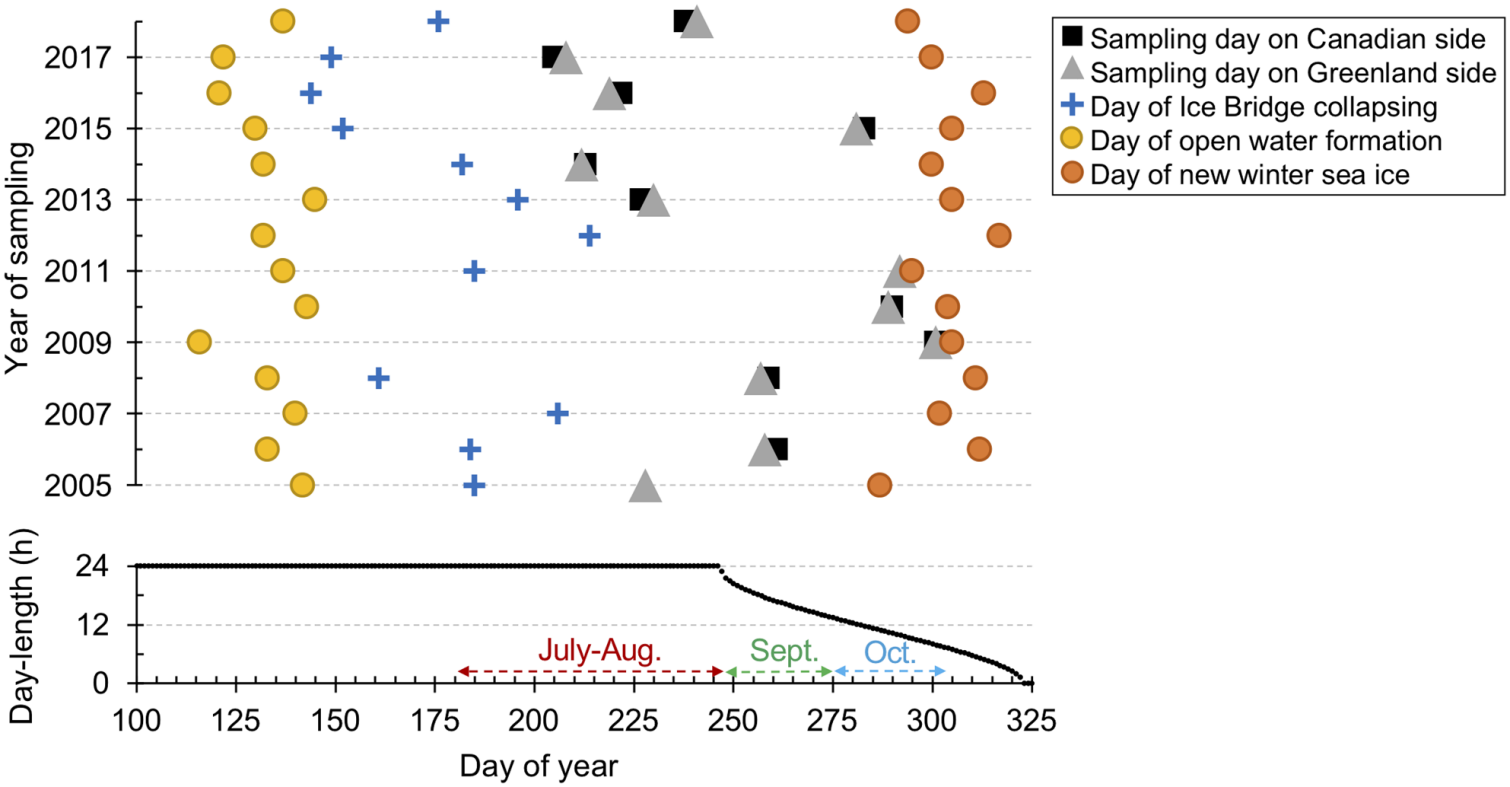
Key events by day of year (Julian date). Upper panel shows dates of selected conditions from 2005 to 2018: the first day of open water forming the polynya (yellow circles) in the North Water; date of the Nares Strait ice bridge collapsing (blue crosses); sampling dates (black squares for Canadian side sampling, grey triangles for the Greenland side) and the date of new winter sea-ice (orange circles) that corresponds to the date when sea-ice covers Nares Strait to the north, and an indication of onset of winter ice conditions (see Supplementary Table S1). The panel below indicates the day-length based on latitude 76.36°N and longitude − 74.01°W over the same time frame.

All samples were collected from open waters and after the ice bridge breached as defined by Dumont et al*.*^20^ (Fig. 1). The date between the opening of the polynya, ice bridge collapse and sample collection varied, providing a range of environmental conditions over time (Fig. 2; Supplementary Table S1). The average depths of the SCM and depths of greatest change in chlorophyll fluorescence (Chl *a*) were similar for both sides of the North Water (Supplementary Table S2). The SCM ranged from 25 to 50 m on the Canadian side (West) and 24 to 80 m on the Greenland side (East), with maximum SCM depths in autumn. The depth of the surface mixed layer from the Brunt-Väisälä frequency, ranged from 12 to 25 m (West) and 7 to 30 m (East), with actual values > 1.0 × 10^−3^; except on the Greenland side in August 2016 and all October dates (2009, 2010, 2011 and 2015). A nitracline occurred above 100 m for all sampling dates.

**Figure 3:**
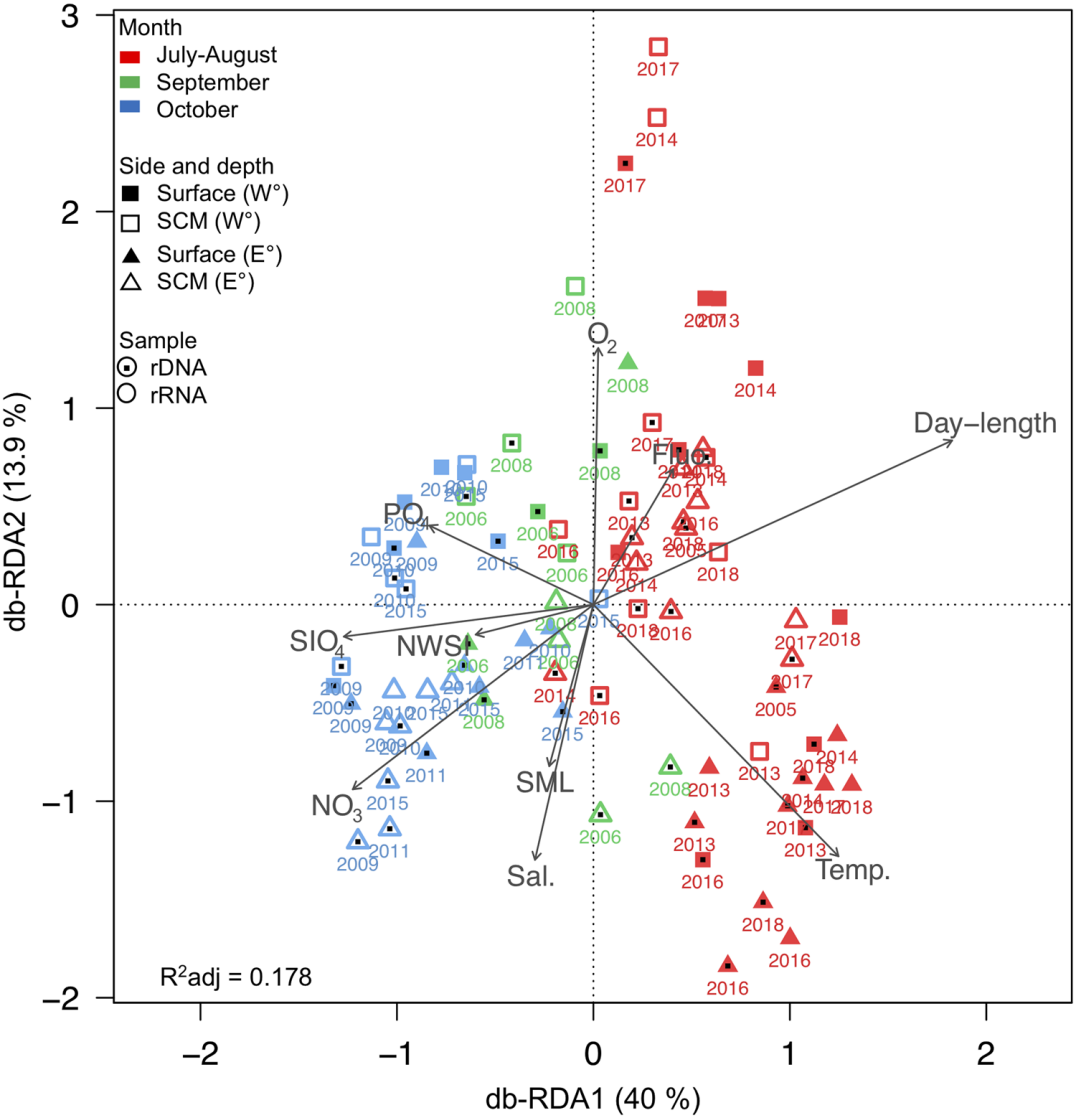
Microbial eukaryotic community clustering using distance-based Redundancy Analysis (db-RDA) with the Bray–Curtis dissimilarity measure. Arrows indicate correlation between environmental parameters and communities assembled from rRNA (open symbols) and rDNA (symbols with interior dots), for the two sides of the North Water: Canada on the Western (W°) and Greenland on the eastern side (E°). Colors indicate month of sampling. Significant factors were water temperature (Temp.), salinity (Sal.), relative Chl *a* fluorescence (Fluo.), nitrate (NO_3_), silicate (SiO_4_) and phosphate (PO_4_) concentration, dissolved oxygen (O_2_), depth of surface mixed layer (SML; Supplementary Table S2), day-length and day of the year for the onset of new winter sea-ice (NWSI) in North Water region (from Fig. 2).

Temperature and day-length differed by month of collection and the communities separated by season (Fig. 3). Other environmental variables: salinity, Chl *a* and nutrient concentrations (Supplementary Tables S3 and S4) differed between July–August, September and October. Additional differences were evident depending on depth category and by side defined as East (Greenland) or West (Canadian). The Welch two-sample *t*-test (Supplementary Table S5) indicated significantly lower surface salinity (*p* < 0.01), as well as nitrate (*p* < 0.05) and phosphate (*p* < 0.01) concentrations compared to the SCM within the two sides. Silicate concentrations were greater at the SCM compared to the surface on both sides but was significant (*p* < 0.01) only on the Canadian side. Water temperature was significantly greater (*p* < 0.05) between depths on the Greenland side (Supplementary Table S5). Taking the two depths combined, we found no significant difference between sides for temperature, Chl *a*, nitrate, silicate or dissolved oxygen. However, salinity was significantly lower on the Canadian versus Greenland side (*p* < 0.001) and phosphate concentrations were significantly greater (*p* < 0.05) on the Canadian side.

### Seasonal differences in the physical conditions

The greatest differences in the physical environment occurred between summer (July–August) and later autumn (October) at the onset of ice formation. A Spearman’s rank correlation (*ρ*) matrix indicated statistically significant co-correlations associated with season (Supplementary Fig. S1). Day-length was positively correlated with temperature but negatively correlated with regional sea-ice and nutrient concentrations. The highest temperature was in 2016 in the summer surface waters on the Greenland side (Supplementary Table S3). The highest values for Chl *a* in surface waters were in July 2014 (2.03 µg Chl *a* L^−1^) and in October 2015 (1.01 µg Chl *a* L^−1^) on the Greenland side. The lowest value was in October 2010 (0.07 µg Chl *a* L^−1^) on both sides. SCM Chl *a* values were greatest in August 2013 (4.14 µg Chl *a* L^−1^) and the minimum value was the October 2010 (0.07 µg Chl *a* L^−1^), both on the Greenland side. Chl *a* was generally higher on the Greenland side during summer at the SCM, especially in 2013 and 2014.

### Strong seasonality of microbial eukaryotes

After the removal of metazoa, bacteria and fungi reads, a total of 500,456 rDNA and 432,213 rRNA reads were retained and clustered into a total of 8,753 OTUs (at 98% similarity) with 7,033 OTUs from rDNA and 7,330 OTUs from rRNA. We failed to detect any trend in communities associated with year of collection (Supplementary Fig. S2). On a community level, distance-based redundancy analysis (db-RDA) placed the majority of October samples together, these were separated from July–August and September samples along the first axis associated with day-length and nutrients. The communities were tested against environmental parameters, and the primary axis (Fig. 3; Supplementary Fig. S3) showed that October communities were associated with the seasonally higher nitrate concentrations (ANOVA: *R*^2^ = 0.01, *F* = 1.84, *p* < 0.05). Summer communities were associated with warmer temperatures (ANOVA: *R*^2^ = 0.05, *F* = 5.7, *p* < 0.001) and longer day-length (ANOVA: *R*^2^ = 0.03, *F* = 3.16, *p* < 0.01). The summer samples were more dispersed along the second axis, associated with temperature and salinity. The second axis of both the db-RDA and constrained correspondence analysis (CCA) plots highlighted oxygen and salinity as further potential environmental filters, with some clustering suggesting separation of West–East communities along with depth category. The Canadian and Greenland sides were examined separately, using PerMANOVA to test for the significance of environmental variables correlated with the combined communities (Supplementary Table S6). The test indicated that on the Canadian side, temperature and salinity were the main drivers of community differences. Temperature was also significant for the Greenland communities but not salinity. However, differences in silicate concentrations between depths were significant on the Greenland side (Supplementary Table S7).

**Figure 4:**
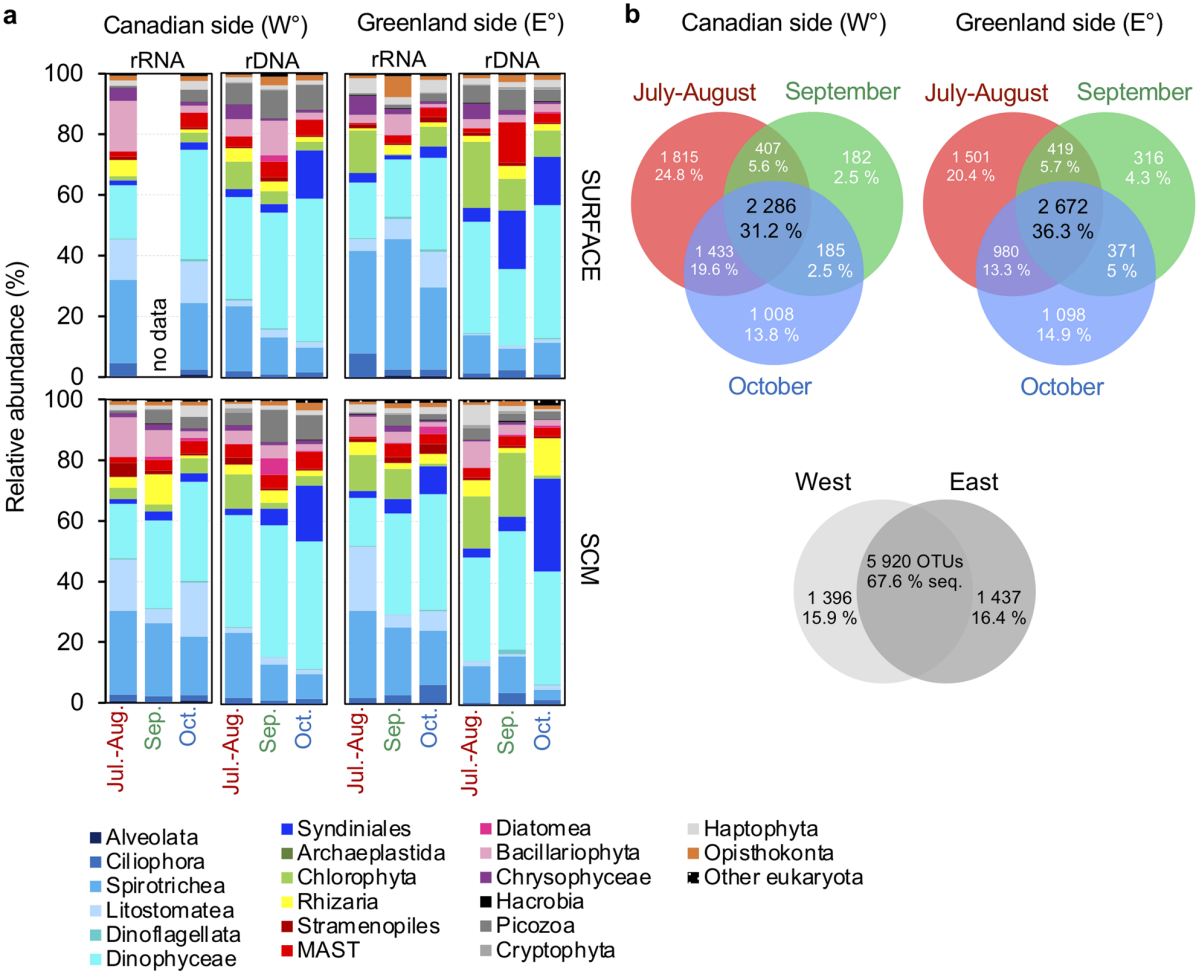
Seasonal and sides comparison of community composition. (**a**) Eukaryotic phytoplankton 18S rRNA gene OTUs community composition. Relative abundance by month of sampling (see dates of sampling in Supplementary Table S1) of both depths and sides. (**b**) Venn diagrams showing the number of unique and shared OTUs detected by 18S rRNA sequencing comparing the three-time frames (July–August, September and October) of the two sides of the North Water from 2005 to 2018. Lower Venn diagram shows overlap of communities between the two sides for all years, with the corresponding percentage of the total OTUs in combined rRNA and rDNA surveys.

The Non-Metric multiDimensional Scaling (NMDS) plot based only on communities, separated the samples by season but added more weight to three 2018 samples (Supplementary Fig. S4). The generally greater dispersion of the summer samples was evident in seasonal Venn diagrams of OTUs from the two sides (Fig. 4b). There were more side specific OTUs in July–August (West: ca. 25%; East: 20%) compared to the October samples with less than 15% for either side. Examining all samples combined, we found ca. 16% of OTUs were unique to one side or the other, with ca. 68% shared.

**Figure 5:**
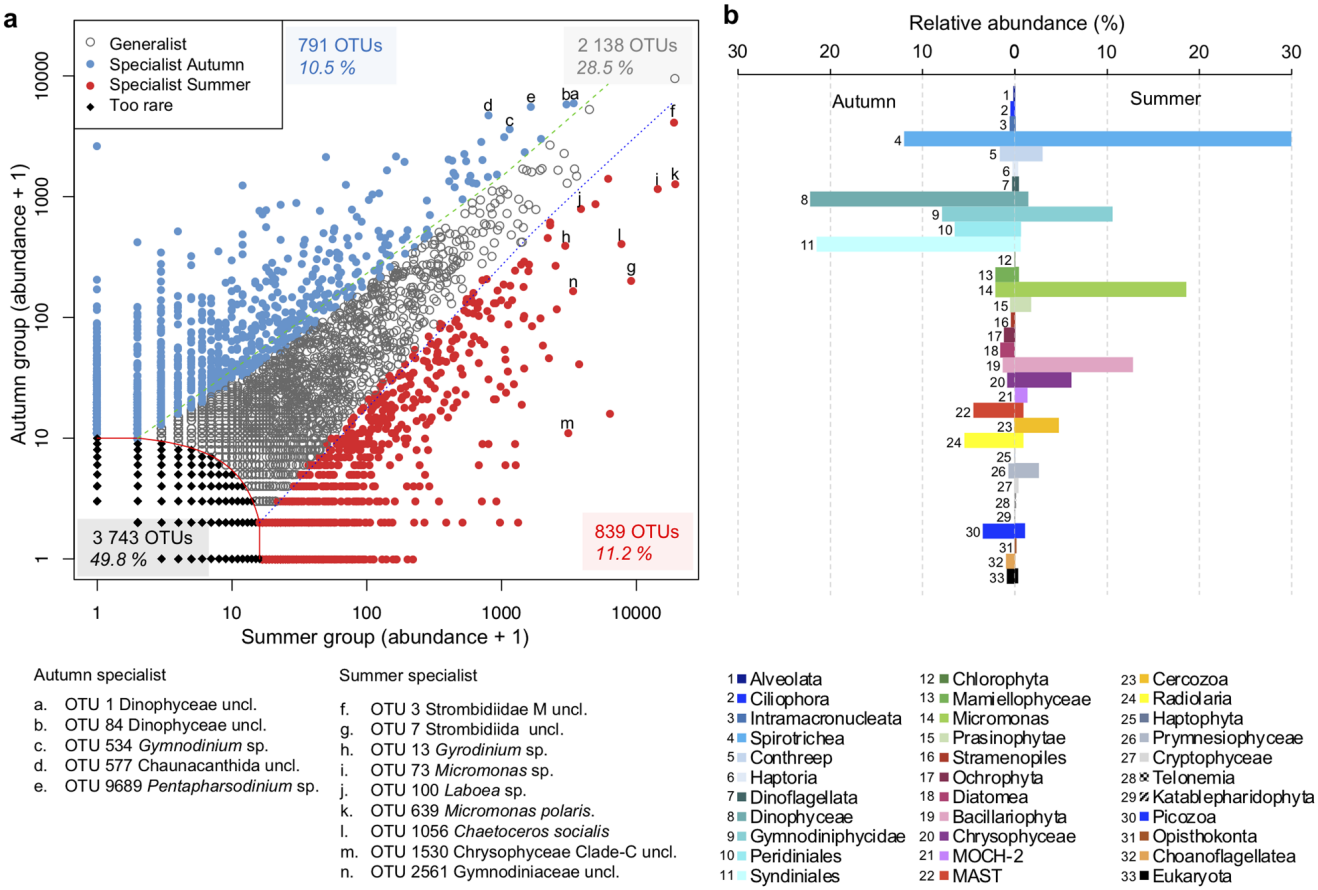
Specialist taxa in summer versus autumn inferred using the CLAM test (see main text). (**a**) The CLAM test plot of all OTUs in summer (July–August, n = 42) and autumn samples (October, n = 28) from both sides, both depths (surface and SCM), from both rRNA and rDNA datasets of all years (2005–2018), excluding 2006 and 2008 from September samples. Note that OTUs with < 10 reads were too rare to be classified. Percentage of total OTUs following the color code. The points marked with letters indicate the position of “top” seasonal indicator OTUs (see Fig. 6). (**b**) Relative abundance of the major groups in summer and autumn (number of reads), legend correspondence is given by both colors and numbers.

Since season was the major factor separating samples, we carried out a pairwise CLAM (see methods) test of July–August (summer OTUs) and October (autumn OTUs) to identify seasonal specialists (Fig. 5a). The test indicated that 11.2% of the OTUs could be considered summer specialists and 10.5% as autumn specialists. Ciliates, mostly in the Spirotrichea and half of one category of dinoflagellate (Gymnodiniales) OTUs were summer specialists. Other summer specialists were *Micromonas* spp. and diatoms (Bacillariophyceae), with each accounting for > 10% of all summer specialists. In autumn, the highest proportions of specialist OTUs were predominantly dinoflagellates with ca. one quarter of the Gymnodiniales and higher proportions of unclassified Dinophyceae, Peridiniales and Syndinales (Fig. 5b).

We further compared all OTUs to determine if there were specialists associated with either side of the North Water (Supplementary Fig. S5). In this analysis, 41% were generalists and 5.6% were specialist of one side or the other. Similarly, there were only a small percentage of OTUs that were classified as either surface (4%) or SCM specialists (4.2%); (Supplementary Fig. S6). All CLAM tests (season, side and depth comparisons) indicated that nearly 50% of OTUs were too rare to be considered as either specialist or generalist. As a check on the viability bias among the OTUs, we also compared taxa that were more represented in the rRNA libraries compared to rDNA. The CLAM test classified ca. 13% of OTUs as specialists of rRNA and 6.5% specialists of rDNA (Supplementary Fig. S7). Such bias suggests different life cycle stages for the taxa, with the rRNA specialists in more active growth stages compared to rDNA specialists, but this conjecture remains to be tested.

### Finer taxonomy of top OTUs

**Figure 6:**
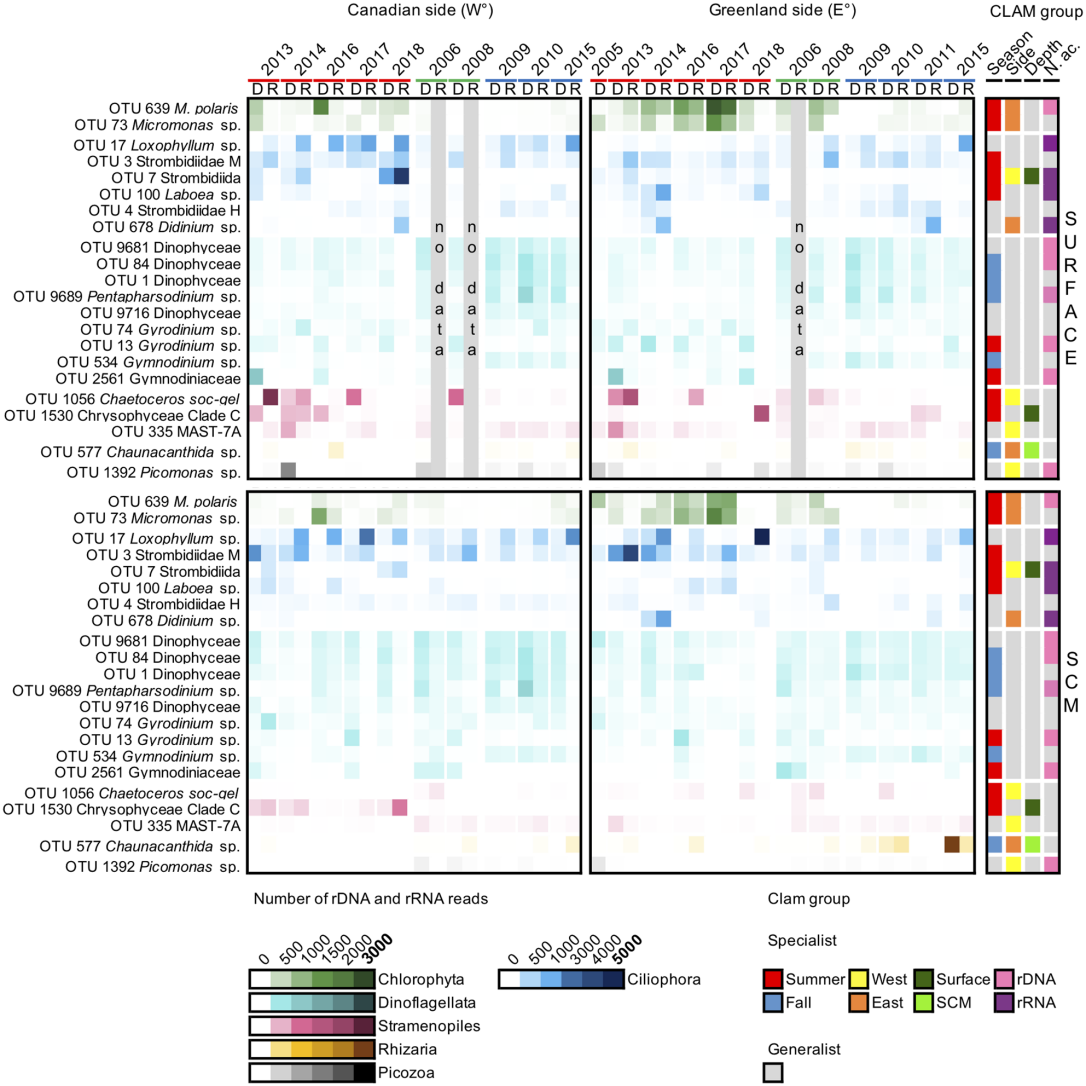
Proportion of reads from the 22 “top” OTUs defined as having reads accounting for > 0.5% of the entire community. Samples are ordered chronologically for each season (July–August: 2005, 2013–2014, 2016–2018; September: 2006 and 2008; October: 2009–2011 and 2015). The heatmap shows the rRNA (R) and rDNA (D) library results (upper axis). Taxa are ordered and color coded by major high-level classification (approximately equivalent to phyla in botanical nomenclature): from most to least abundant in terms of reads within the phyla (see Fig. 7 for identification of the OTUs). The color intensity in the heatmap reflects the number of reads of each taxon with darker intensities indicating higher read counts, note the different scale for Ciliophora. Based on the results of CLAM test (Fig. 5; Supplementary Figs. S5, S6 and S7), the corner right bars indicate specialist taxa. Color coded for specificity to a season, a region, a depth or to nucleic acid source. Grey in all bars indicate non-specialist taxa for the category.

**Figure 7:**
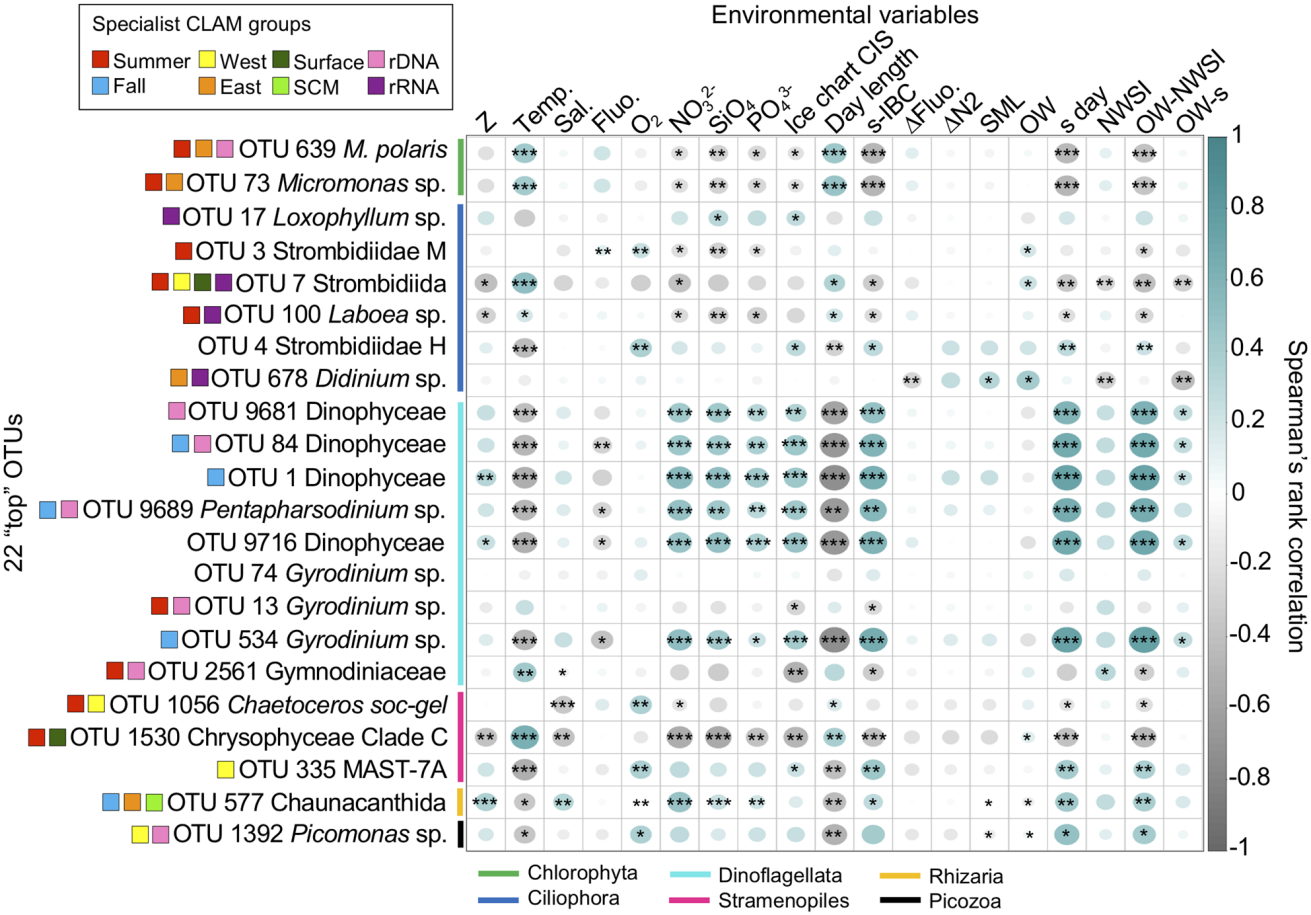
Spearman’s rank correlation (*ρ*) matrices between environmental variables and the 22 top OTUs (reads > 0.5% of the entire community) from both rDNA and rRNA reads. Spearman correlation coefficient values are represented by the size of the circles (larger for high coefficients; smaller for low coefficients) and color (blue – positive; grey – negative), empty squares indicate no correlation. Asterisks within circles represent the significance level *p*-value from Spearman’s *Rho* (*p* < 0.05 *, *p* < 0.01 **, *p* < 0.001 ***)*.* Environmental variables: depth of sampling (Z), water temperature (Temp.), salinity (Sal.), Chl *a* fluorescence (Fluo.), dissolved oxygen (O_2_), nitrate (NO_3_^2−^), silicate (SiO_4_), phosphate (PO_4_^3−^). Also given are values from the visual analysis of ice charts produced by the Canadian Ice Service (CIS): day-length (from Fig. 2); days between sampling (s) date and ice bridge collapsing (s-IBC); depth of maximum mean vertical gradient in fluorescence, dFluo./dz (ΔFluo.); pycnocline is the depth of Brunt-Väisälä frequency maximum (ΔN^2^); depth of the Surface Mixed Layer (SML); day of the year of when the polynya opened as open water (OW); day of the year when the samples were collected (s day); day of the year when new winter sea ice (NWSI) occurred; total days between OW and NWSI (OW-NWSI); days from the beginning of OW to sampling (OW-s).

To gain insight into the ecology of the region’s dominant taxa, we identified “top” OTUs that accounted for at least 0.5% of all reads in the study. Overall, only 22 OTUs met the criteria (Figs. 6 and 7). Dinophyceae accounted for nine of the top OTUs. Four of these nine were assigned to Gymnodiniales, including OTU-74 classified as *Gyrodinium spirale* (PR^2^ database), but was distant when placed in the RAxML tree using *pplacer* (Supplementary Fig. S8). In addition, *pplacer* assigned two of the OTUs (OTU-13 and -2561) to the *Gyrodinium rubrum*–*helveticum* clade. One unclassified OTU-1 aligned within Kareniaceae and the other three unclassified dinoflagellate OTUs (OTU-84, -9681 and -9716) grouped separately with other uncultivated sequences from offshore marine waters, including from the Arctic and sequences from 2500 m in the East Pacific Rise (KC488435 and KJ757193 respectively). The “*Gymnodinium*” (OTU-534) was within a clade that also included radiolarian symbionts and the kleptoplastidic *Lepidinium virde* that maintains green algal chloroplasts. The final dinoflagellate (OTU-9689) was assigned to the Thoracospaeracea, in the genus *Pentapharsodinium* (by PR^2^) but was put into a clade of *Scrippsiella* sequences using *pplacer*. Given their ubiquity and placement in the 18S rRNA gene tree, the four autumn specialists (OTU-1, -84, -534 and -9689) fit with the notion of being arctic indicators or sentinels. Two dinoflagellates were summer specialists (OTU-13 and -2561). The three others were considered generalists and none of these top dinoflagellate OTUs were classified as specialist of a particular depth or side of the North Water (Figs. 6 and 7).

Ciliate OTUs accounted for six of the top OTUs. Four were Strombidiida, with three assigned to environmental Strombidiidae categories (OTU-3, -4 and -7) and one to the genus *Laboea* in the Tontoniida (OTU-100). The other two were Lithostomatea and included *Didinium* (OTU-678) and *Loxophylum* (OTU-17). Three of these were summer specialists (OTU-3, -7 and -100) and the other three seasonal generalists. Four were classed as rRNA specialists. The Strombidiida OTU-7 appeared as highly specialized to the surface summer on the Canadian side (Figs. 5a and 6; Supplementary Figs. S5 and S6).

The diatom *Chaetoceros* (OTU-1056) was among the top OTUs and assigned to *C. socialis* by PR^2^ but had a nearest BLAST (Supplementary Table S8) match to *C. gelidus* (strain RCC2271) from the Beaufort Sea. One chrysophyte (OTU-1530) could not be identified to a genus but was within Chrysophyceae clade C, which includes most *Ochromonas* spp. and *Dinobryon*. These two OTUs were considered as summer specialists, with *Chaetoceros* a Canadian side specialist and the chrysophyte a surface specialist (Fig. 6).

Two other top OTUs belonging to photosynthetic taxa were both *Micromonas*, with *M. polaris* (OTU-639) accounting for the highest proportion of total reads ca. 2.5% and a second *Micromonas* (OTU-73), which was related to the clade B subarctic (by PR^2^). Other *Micromonas* OTUs were recovered in much smaller percentages (Supplementary Fig. S9). The CLAM test classified both top *Micromonas* OTUs as summer and Greenland side specialists with OTU-639 as a rDNA specialist (Fig. 6).

The remaining three top OTUs were heterotrophic taxa. OTU-335 was classified as a heterokont in the MAST-7A clade and OTU-577 was in the Acantharia (Rhizaria) in the order Chaunacanthida, with closest matches to other environmental sequences and 98.6% similar to *Heteracon* sp. Ant-21 (Supplementary Table S8). OTU-1392 was classified as a Picozoa. The CLAM test classified MAST-7A and *Picomonas* sp. as Canadian side specialists and Chaunacanthida as an autumn SCM Greenland side specialist (Fig. 6; Supplementary Fig. S5).

### Environmental and taxon correlation

A Spearman’s rank correlation (*ρ*) matrix comparing top OTUs with environmental factors (Fig. 7) and each other (Supplementary Fig. S10) indicated the positive correlation between day-length and temperature for the two *Micromonas* spp. and the chrysophyte. Six dinoflagellate top OTUs (OTU-1, -84, -534, -9681, -9689 and -9716) were negatively correlated with temperature and positively correlated with nutrient concentrations. These six were co-correlated with each other (Supplementary Fig. S10) and with the occurrence of *Picomonas* and MAST-7A. For the most part, the top ciliate OTUs were negatively correlated with the six co-correlated dinoflagellates.

Overall, this multiyear study highlighted the strong seasonality in the microbial eukaryotic communities with a distinct October community and more diverse and varied summer communities. Water temperature was a major variable influencing the microbial eukaryotic community. The surface temperatures in October on both sides were close to the freezing point of seawater (− 1.78 °C; Supplementary Table S3) and appeared to favor dinoflagellates (Figs. 4a and 5b). Temperature co-varied with other seasonal indicators, such as day-length and time since the initial opening of the polynya (open water) in a particular year, as well as nutrient concentrations (Supplementary Fig. S3). Higher nutrients in surface samples were associated with lower temperatures in October suggesting deeper waters entrained into the surface due to weaker stratification (Supplementary Table S2).

## Discussion

In total, the autumn dinoflagellates accounted for around 75% of reads in our study, compared to ~ 50% of summer reads (Fig. 4a). None of the top autumn OTUs were diatoms, consistent with diatoms being limited by low autumn light availability and non-diatoms dominating under lower light conditions^21^. Autumn Chl *a* concentrations remained low in the surface and SCM, suggesting that the dinoflagellates were not overly reliant on photosynthesis. About half of all described dinoflagellates are heterotrophic with the vast majority of photosynthetic dinoflagellates able to capture prey^22^, with mixotrophy reported in Arctic dinoflagellates^23^. In accordance with this autumn community being on the mixotrophic–heterotrophic continuum, the three other autumn top OTUs belonged to heterotrophic groups (MAST-7A, Chaunacanthida and *Picomonas*). These were all negatively correlated with temperature and positively correlated with sampling just prior to new winter sea-ice formation (Fig. 7).

The top summer specialist dinoflagellates (OTU-13 and -2561) were both in the *Gyrodinium helveticum–rubrum* clade that has been reported from other regions of the Arctic^1,24^. In the Beaufort Sea, the clade was common both in summer and autumn mesopelagic samples^25^, which may have been due to sinking aggregates or an ability to exploit wide spectrum of conditions.

While the October alveolate community showed a high proportion of dinoflagellates, the highest proportion of reads in summer belonged to ciliates. The majority of summer ciliates were in the Spirotrichea, which includes the order Strombidiida (PR^2^ classification), which is more or less synonymous with the Order Oligotrichia (Silva database). Oligotrichia are often reported to be kleptoplastidic retaining chloroplasts from prey species to facilitate mixotrophy. In particular, the summer specialist *Laboea* (OTU-100) favors the retention of *Micromonas* chloroplasts and contributes to primary productivity in temperate seas^26^. It was also previously reported from summer North Water samples^17^ and associated with longer day-length in the Beaufort Sea^27^, in keeping with it being a summer specialist.

Year to year variation in species was evident with the highest proportions of total ciliate reads in summer of 2018, when ciliates dominated communities on both sides (Supplementary Figs. S2 and S4). *Loxophyllum* (OTU-17) had exceptionally high read proportions on the Greenland side at the SCM, suggesting a possible ciliate “bloom”, defined as higher-than-normal biomass within a given taxon. The “bloom” appeared widespread and while read counts were higher on the Greenland side, *Loxophyllum* was recorded on the Canadian side (Fig. 6). Ciliate blooms other than *Mesodinium rubrum*^28^ are not often reported but given the dominance of this single taxon it should be investigated in the Arctic.

In contrast to autumn, top summer OTUs included chrysophytes, along with diatoms and chlorophytes that are strictly photosynthetic phytoplankton (Fig. 6). The chlorophyte genus *Micromonas*, in particular *M. polaris*, is a key component of Arctic marine food webs^29^ and is frequently reported from summer nutrient-depleted waters of the Arctic Ocean. *M. polaris* has a pan-Arctic distribution^29,30^, is adapted to cold temperatures and can persist under low-light regimes, with maximum growth rates around 6°C^29^. Although *M. polaris* is found all year round^25,31^, it tends to decrease during the winter^24,31^ in accord with being a summer specialist. A second putative species or ecotype of *Micromonas* (OTU-73), was also revealed with the massive sequencing effort over the 12 years. In addition, at least in the Arctic where *Micromonas* is the dominant picophytoplankton, viral interactions could also play a role in driving population dynamics and seasonality^32,33^. The two dominant *Micromonas* OTUs were both related to cold water isolates. OTU-639 was 100% identical to CCMP2099 that was originally isolated from the North Water and OTU-73 was closest to clones from Svalbard and the North Atlantic (Supplementary Table S8 and Fig. S9).

For our multi-year study, although temperatures never exceeded 4.5 °C in any year, both *Micromonas* top OTUs were correlated with temperature along with longer day-length. Both were classified as specialists on the Greenland side (Figs. 6 and 7; Supplementary Fig. S5), which tended to be warmer in summer, but neither were ever absent from the Canadian side. Overall, given the very small temperature changes influencing relative abundance and occurrence, it seems that the Arctic *M. polaris* is truly a sentinel species in the Arctic^34^.

The spring bloom in the Arctic is generally dominated by larger centric, especially Thalassiosiroid diatoms^35,36^, followed by species of hyalochaete *Chaetoceros* in the Mediophyceae. In particular, biomass is dominated by colonies of the pan-Arctic *C. gelidus*, which was previously classified with *C. socialis*^2^. *C. gelidus* is widely distributed in the Arctic and can remain abundant into July, August and even September, despite diminishing nutrient concentrations over the course of the season^18,37^. The persistence in the North Water is attributed to nutrient input from Pacific origin water from Nares Strait and the Canadian Arctic Archipelago via Jones Sound^18^. Because these conditions favor this lightly silicified *Chaetoceros*, the species is critical in supporting the overall biological richness of the polynya^37^.

The top *Chaetoceros* (OTU-1056) was automatically classified as *C. socialis* but was most similar to *C. gelidus* albeit with nucleotide differences compared to the type species from the Beaufort Sea. A true North Atlantic *C. socialis* could also occur in the region, due to the input of Atlantic Water in the north flowing West Greenland Current. Alternatively, OTU-1056 could represents a distinct regional ecotype within a larger *C. socialis-gelidus* species complex. Over the 12 years period of our study, the same OTU-1056 was often abundant during summer months, especially on the more Arctic water influenced Canadian side (Figs. 5b and 6), suggesting that this is a North Water ecotype and not attributable to Atlantification^38^ of the northern North Water. The significant correlation between lower salinity and the OTU was consistent with fresher Arctic modified waters on the Canadian side (Fig. 7).

The filtration strategy and use of the small size (0.2–3 µm) fraction followed previous molecular surveys^1,39^ to better detect small eukaryotic cells. However, many of the 18S rRNA gene sequences belonged to presumably larger protists, mostly dinoflagellates and ciliates (Fig. 4; Supplementary Fig. S2), as previously reported using the same approach^27^. Reasons for this have been discussed elsewhere and deformation of flexible-walled cells and cell breakage during filtration would lead to reads from larger cells in the small fraction. The high read counts in the rDNA for dinoflagellates, would reflect the larger genomes and multiple rRNA gene copy numbers. However, we note that a high proportion of dinoflagellates was reported in a recent study of Arctic phytoplankton with dinoflagellates accounting for 11–45% of phytoplankton across the Arctic, consistent with dinoflagellates as important contributors to Arctic microplankton biodiversity^40^. Ciliates were “rRNA” specialists, which would be consistent with high numbers of ribosomes^41^. Dinoflagellates and ciliates are routinely reported as mostly unidentified species in Baffin Bay, with dinoflagellate biomass variable^14^. And both groups were abundant and reported in the 1998 North Water Polynya study. Overall, our molecular approach enabled us to identify seasonal and spatial trends at a much more refined taxonomic level than is possible using routine microscopy, where species assignation can be subjective and dependent on the experience and expertise of individual researchers.

In summer, the differences between the two sides of the North Water communities reflected the differences in hydrography, dominant surface currents and water mass distributions. All of these factors influenced nutrient concentrations and salinity that were the main explanatory variables separating surface and SCM communities (Fig. 3; Supplementary Fig. S3). In particular, salinity had a strong influence on Greenland side, where warm salty Atlantic waters are found at depth^4^ beneath fresher surface waters influenced by ice melt. The increased stratification promoted the formation of an SCM^14^. However, only one of the top OTUs was an SCM specialist (Chaunacanthida, OTU-577) and only two (Strombidiida, OTU-7 and Chrysophyceae, OTU-1530) were surface specialists, suggesting that the majority of the higher abundance OTUs do relatively well throughout the euphotic zone. There were differences between the two sides, with each side having specialist top taxa (Fig. 6). The picoplanktonic *Micromonas* along with the Chaunacanthida and the ciliate *Didinium* (OTU-678) were most often on the Greenland side and associated with warmer temperatures. In contrast, the western specialists included *Chaetoceros* (OTU-1056), a Strombidiida (OTU-7) and two picoplanktonic heterotrophs (MAST-7A, OTU-335 and *Picozoa*, OTU-1392), suggesting a different microbial food webs operating on the two sides.

Day-length and light availability are critical at high latitudes where day-length runs from 0 to 24 h. In our limited seasonal sampling, where we were only able to collect samples during the ice-free summer and late autumn, the July–August samples were collected when the sun was above the horizon 24 h a day. Here, *Micromonas* spp. were correlated with longer day-length and lower nutrients **(**Fig. 7) that are characteristic of post-spring-bloom conditions. Light was the primary limiting resource in October, and we found little evidence of increased Chl *a* in October even though nutrient levels were higher. The sun sets below the horizon starting in early September at this latitude and following the autumnal equinox, day-length shortens rapidly, and the shortest day sampled was less than 8 h of direct sunlight (Fig. 2; Supplementary Table S1). The lack of a late season bloom contrasts with the expectation that autumnal blooms will occur due to an extended open water season because of a later ice formation, with the phytoplankton exploiting the nutrients supplied by convective mixing^16^. In this case, the high latitude of the North Water may have precluded an October bloom as diatoms would have been light-limited due to low sun angle and the deeper mixed layer^7^.

To date, evidence of fall blooms or increased Chl *a* biomass has been reported for inflow shelfs and linked to climate change^42^. Ours is the first multiyear study of an outflow shelf to our knowledge, and it remains to be seen if outflow shelves are generally less susceptible to fall blooms. A caveat for the lack of change over the 12 years could be that the series began after major changes had already occurred. Our results highlight the need for further in situ studies and monitoring along with well-designed paleoceanography studies aimed at recovering planktonic DNA in the sediments. The differences in relative abundance of OTUs was mostly associated with season and somewhat by geography, depth and nucleic acid source. In summer, sentinel species such as *Micromonas polaris* and *Chaetoceros socialis* (*C. gelidus*) were favored by slightly warmer surface temperatures and nutrient-poor waters at the end of summer. Dinoflagellates, especially from an uncultured oceanic Dinophyceae group were associated with autumn and correlated with cold and nutrient-rich waters, at least 3 OTUs were placed on long branches compared to other uncultured sequences available suggesting that they could be typical of current conditions and sentinels that should be monitored to detect ecosystem change. Although more sampling from other regions is needed, autumn phytoplankton in the Arctic may be dinoflagellate dominated and not ecologically equivalent to diatom dominated spring and early summer blooms.

## Materials and methods

### Study site

Data on ice dynamics of the region (Baffin Bay and Nares Strait) were extracted from satellite images from National Snow and Ice Data Center (NSIDC) and from NASA Earth Observatory (SMMR, SSM/I and SSMIS sensors) from 2005 to 2018^43^. The beginning of open water (OW) and the start of the new winter sea-ice (NWSI) period were determined as in Marchese et al*.*^10^. The duration of the OW season was defined as number of days between opening of the North Water polynya and NWSI, when ice reformed over the region. The date of ice bridge collapse (IBC) in Nares Strait was determined from a visual analysis of ice charts produced by the Canadian Ice Service and images from NSIDC and NASA consistent with earlier studies^14,44^.

### Samples collection

We analysed a total of 50 samples that had been collected onboard the research icebreaker CCGS *Amundsen* between 2005 and 2018 during multidisciplinary missions, associated with ArcticNet. The missions were carried out nearly annually along West–East transects on a line that varied from 76.20°N to 76.58°N, depending on ice and other mission constraints (Fig. 1; Supplementary Table S1). A primary goal of our study was to examine variability of the two sides of the northern North Water ecological region between Ellesmere Island, Nunavut, Canada and Greenland for which we selected samples from the westernmost and easternmost stations (Stn) of the transect. Because of mission constraints, the samples were collected in different seasons in the different years (Fig. 2), specifically at the end of July through August in 2005, 2013, 2014, 2016, 2017 and 2018; September in 2006 and 2008 and October in 2009, 2010, 2011 and 2015. The transect was not carried out in 2007 or 2012 and no samples were available from these two years. The majority of the westernmost samples were from ArcticNet Stn101, which was sampled 2006, 2008, 2013, 2014, 2017 and 2018. However, due to ice conditions preventing the ship reaching Stn101, samples were taken from the next most westerly station (Stn105) in 2009, 2010 and 2016. The easternmost station on the Greenland side, Stn 115, was sampled in all years. We targeted the upper polar mixed layer (surface; 0–15 m) and the SCM detected on the downward cast. This feature normally forms below the polar mixed layer at the top of a halocline (20–80 m).

Physical oceanographic profiles were collected on the downward cast using a rosette system that was equipped with a conductivity, temperature, depth (SBE-911 CTD, Sea-Bird Electronics Inc.) profiler, sensors for chlorophyll fluorescence (Seapoint Sensors Inc.) and relative nitrate from the ultraviolet spectrophotometer (ISUS, Satlantic). All water samples were collected on the upward cast. Nutrient samples were collected every 10 m from the surface to 100 m. The samples for nutrients were taken directly from the 12L Niskin-type bottles mounted on the rosette, filtered through a 0.2 µm polycarbonate filters (PC, AMD Manufacturing) into acid cleaned and rinsed PC tubes and kept at 4 °C until analysis within 4 h of collection. Nitrate + Nitrite, Silicate and Phosphate were analyzed on board using a Bran-Luebbe 3 auto-analyzer^45^.

Water samples for microbial DNA and RNA were collected from the Niskin-type bottles into cleaned acid rinsed carboys. The water was pre-filtered immediately onboard through a 50 µm nylon mesh to remove macrozooplankton. Then sequentially through a 3 µm pore size PC filter and a 0.2 µm Sterivex Unit (Millipore Canada Ltd.) using a peristaltic pump system (Cole-Palmer Company). Prior to 2010, samples for DNA and RNA were filtered separately as described in Terrado et al*.*^46^ (Supplementary Table S9). Briefly, for DNA, lysis buffer was added to the Sterivex and cryovials, which were then frozen at − 80 °C. For RNA, samples were filtered as indicated above except that only 4L of seawater was filtered for each sample and filters were preserved in RLT buffer (Qiagen) with 1% β-mercaptoethanol (Sigma-Aldrich) flash frozen in liquid nitrogen and stored at − 80 °C. After 2011, samples for DNA and RNA were collected on the same filters and preserved by adding 1.8 mL of RNAlater (Ambio, ThermoFisher Scientific) to the Sterivex and cryovials. After 1 h in buffer, the filters were frozen at − 80 °C until processing in the laboratory. In an effort to identify smaller organisms typical of later in the season^14^, we focused the study on 0.2–3 µm fraction, recognizing that larger organisms are captured on the same filters, most likely due to cell breakage.

### Nucleic acid extraction and sequencing

For samples collected in 2005 and 2006, DNA was extracted from the filters using an equal volume of phenol–chloroform-isoamyl alcohol (Supplementary Table S9). For samples collected from 2008 to 2011, DNA was extracted from the filters as described in Terrado et al*.*^46^ based on a salt extraction method. The RNA samples from 2005 to 2011 were extracted using the RNAEasy Micro Kit (Qiagen). After 2013, DNA and RNA were extracted from the same filters using the All-Prep DNA/RNA Mini Kit (Qiagen) as previously described^47^. For all years, conversion of RNA to cDNA was carried out using the High-Capacity Reverse Transcription Kit (Applied Biosystems) following the manufacturer’s suggestions. The V4 region of 18S rRNA in both DNA and cDNA samples was amplified using eukaryotic specific forward primers (E572F) and reverse primers (E1009R)^48^ coupled with a MiSeq specific linking primer. Unique pairs of barcodes were added to the sample amplicons using the TruSeq and Nextera (Illumina) barcode sets in a nested PCR under conditions previously described^48^. The PCR products were purified using the Axygen PCR cleanup kit and quantified spectrophotometrically with the Nanodrop 1000 (ThermoFisher Scientific). For each run, equimolar concentrations of amplicons from each sample were pooled for multiplex sequencing using the MiSeq at the Plateforme d’Analyses Génomiques (IBIS, Université Laval, Québec, Canada).

### Sequence data processing and taxonomic classification

Resulting reads were quality filtered, checked and de-replicated using BBMerge (v.37.36)^49^. Putative chimeric sequences and singletons were removed using VSEARCH (v.2.5)^50^. The retained reads were clustered into Operational Taxonomic Units (OTU) at 98% similarity using USEARCH (v.10.0.240)^51^ and mothur (v.1.41.1)^52^ as in Comeau et al*.*^53^. OTUs were assigned a taxonomy using the Silva Reference Database (v.132)^54^ and the Protist Ribosomal Reference (PR^2^) database (v.4.11)^55^; (Supplementary Table S10). To facilitate diversity comparisons of single-celled microbial eukaryotes, the OTU matrix was filtered to eliminate metazoan (296 reads in total), fungi (30 reads) and Streptophyta (73 reads), then rarefied, based on the sample with the fewest reads (11,374 reads) using QIIME^56^.

The 22 “top” OTUs (with > 0.5% of total reads from the entire data set) were selected to identify species specific trends. The 22 selected taxa belonged to higher-level taxonomic groups: Ciliophora, Dinoflagellata, Chlorophyta, Picozoa, Rhizaria, Chrysophyta, Bacillariophyta and the heterotrophic Marine Stramenopiles (MASTs). The top dinoflagellate OTUs were taxonomically placed onto the reference phylogenetic tree using the Evolutionary Placement Algorithm (EPA) of RAxML^57^. The reference tree was constructed from curated full-length 18S rRNA gene sequences of the specific groups. Sequences were aligned using MUSCLE (v.8.1.13)^58^ and phylogenetic tree constructed using randomized accelerated maximum likelihood (RAxML v.8.2.11, 1000 bootstrap replicates, model GTRGAMMA)^59^.

### Calculations and statistics analyses

Stratification in the upper 100 m was inferred using two methods (Supplementary Table S2). First the surface mixed layer depth was determined by the depth^60^ at which the gradient in density between two successive depths was > 0.01 kg m^−4^. A second method relied on the Brunt-Väisälä (or buoyancy – N^2^) frequency in the upper water column, defined as the depth when N^2^ was the greatest in the upper waters^11^. The Nitracline (∆NO_3_^−^) was defined as depth where the vertical gradient in NO_3_^−^ concentration (dNO_3_^−^/dz) was greatest^11^. Day-length was calculated from nrc-cnrc.gc.ca and based on latitude 76.36°N and longitude − 74.01°W. The pairwise Multinomial Species Classification Method (CLAM)^61^ test was used to classify each OTU into a category: generalist, specialist of one habitat or of the second habitat or too rare. To separate samples we used *clamtest()* function in R (v.3.4.2) with a *p*-value of 0.01 and a coverage limit of 10 sequences as a rarity threshold. We applied the CLAM test between 4 different groups of samples: rDNA or rRNA; West (Canadian side) or East (Greenland side); surface or SCM and summer (July and August) or autumn (October).

NMDS was computed using the *metaMDS()* function to compare similarity between samples. Distance-Based Redundancy Analysis (db-RDA) and Constrained Correspondence Analysis (CCA) were computed using *dbrda()* and *cca()* functions, to discriminate the different sampling stations according to the environmental variables. The independent parameters that best explained variability in the db-RDA and CCA were selected using *ordiR2step()* and *envfit()* functions, by automatic forward selection, which selects variables to build optimal model with the highest adjusted coefficient determination. Non-Parametric Multivariate Analysis of Variance (NP-MANOVA) and Analysis of Similarity (ANOSIM) were used to test differences in composition between seasons, sides and depths. Permutational Multivariate Analyses of Variance (PerMANOVA) was conducted to analyze the correlations between environmental factors and community changes, using *adonis()* function. To test differences in environmental parameters between stations and depths, Welsh two-sample *t*-tests, verified with a non-parametric Kruskal–Wallis One-Way Analysis of Variance on ranks were used.

## Supplementary Information

Supplementary Information 1.

Supplementary Information 2.

Supplementary Information 3.

Supplementary Information 4.

Supplementary Information 5.

## Data Availability

All amplicon results are in the NCBI GenBank Sequence Read Archive (SRA) under BioProject PRJNA383398 (GenBank: SRX2745641, SRX2745642, SRX2745625 and SRX2745626) and PRJNA662595 (GenBank: SRR12643378 to SRR12643456). Oceanographic data is available from Amundsen Science: Amundsen Science Data Collection. [2005–2018]. Processed data. Version 1. Archived at www.polardata.ca, Canadian Cryospheric Information Network (CCIN), Waterloo, Canada. https://doi.org/10.5884/12713. Accessed on [July 2019].

## Code availability

Computer codes and scripts are available from the corresponding authors upon request.
