# Supplementary material for: A decadal perspective on north water microbial eukaryotes as Arctic Ocean sentinels
Source: Sci Rep. 2021 Apr 16;11:8413. doi: 10.1038/s41598-021-87906-4 (PMC8052464; doi:10.1038/s41598-021-87906-4)
Supplement: Supplementary file 5 — Supplementary Information 5. [file 41598_2021_87906_MOESM5_ESM.docx]

**Supplementary Tables**

**Supplementary Table S1.** Supporting information for Fig. 2 (main text). Stations sampled during ArcticNet missions onboard CCGS Amundsen in the North Water from 2005 to 2018 for the Greenland side (East, Stn115) and 2005, 2007, 2011 to 2012 for the Canadian side (West, Stn101 and Stn105). Day-length was based on latitude 76.36°N and longitude -74.01°W (https://www.nrc-cnrc.gc.ca/). Open water (OW) corresponds to the date when the polynya opened (mm/dd), new winter sea-ice (NWSI) corresponds to the date when the polynya was covered in ice, the ice bridge collapsed (IBC) is the date when ice from the north began moving south, and ‘s day’ is the day of the year when the samples were collected.

**Supplementary Table S2.** Water column details on depth for pycnocline, nitracline, surface mixed layer, maximum depth of Chlorophyll *a* fluorescence and subsurface of chlorophyll maximum depth (SCM) at the time of sampling from 2005 to 2018. Acronyms: depth of maximum mean vertical gradient in fluorescence, dFluo./dz, (ΔFluo.); N^2^ is the highest value of Brunt-Väisälä Frequency (10^-3^ $\times$ s^-2^); nitracline is the depth of maximum of vertical gradient in NO_3_^2-^ concentration, dNO_3_^2-^/dz (ΔNO_3_^2-^); pycnocline is the depth of Brunt-Väisälä frequency maximum (ΔN^2^) and depth of the surface mixed layer (SML). All values were calculated between surface to 100m deep, except for value in parenthesis, which was calculated from surface to bottom depth.

**Supplementary Table S3**. Values for temperature, salinity and Chlorophyll *a* fluorescence at each sampling depth: surface (Surf.), subsurface chlorophyll maximum (SCM) from 2005 to 2018. Colors indicate the lowest (blue) and highest (red) values for summer and autumn samples of each side. Sampling dates for the separate sides are arranged by month categories based on Fig. 3 db-RDA (main text) result.

**Supplementary Table S4.** Nutrient concentrations at each depth: surface (Surf.), subsurface chlorophyll maximum (SCM) of sampling from both sides of the North Water from 2005 to 2018. Colors indicate the lowest (blue) and highest (red) values for summer and autumn samples from each side. Sampling dates for the separate sides are arranged by month categories based on Fig. 3 db-RDA (main text) result.

**Supplementary Table S5.** Two-way comparisons between environmental parameters from all years sampled (2005-2018) using Welch unequal variances *t*-test. First column shows between depth categories of the Canadian side (West) and second column shows between depth categories of the Greenland side (East). Last column shows both depths combined for a comparison between the Canadian side and the Greenland side.

**Supplementary Table S6**. PerMANOVA test comparing environmental factors of surface and of subsurface chlorophyll maximum (SCM) of all samples taken of the North Water. Permutational multivariate analyses of variation using distance matrices of environmental factors and community changes. Temperature (Temp.), salinity (Sal.), Chl *a* fluorescence (Fluo.), F model (F) and Pr(>F) is *p*-value based on Monte Carlo random draws.

**Supplementary Table S7.** PerMANOVA test comparing environmental factors of all depths for each side of the North Water. Permutational multivariate analyses of variation using distance matrices of environmental factors and community changes (acronyms as in Supplementary Table S6).

**Supplementary Table S8.** Closest BLAST (basic local alignment search tool) match to the 22 top OTUs of phylum Dinoflagellata, Ciliophora, Chlorophyta, Ochrophyta, Rhizaria, Hacrobia and stramenopiles.

**Supplementary Table S9.** Water filtration and samples extraction methods used throughout the years from 2005 to 2018.

**Supplementary Table S10.** Number of reads observed for each Operational Taxonomic Unit (OTU) in each sample (n = 83) from both acid nucleic sources (rDNA and rRNA). Taxonomy at hierarchical levels is given following the Silva Reference database (v.132) and the Protist Ribosomal Reference (PR^2^) database (v.4.11).

**Supplementary Figures list**

**Supplementary Fig. S1. Spearman’s rank correlation (*ρ*) matrix between environmental parameters from all samples**. Spearman correlation coefficients are represented by the size (the larger the circles the greater the value of the coefficients) also indicated by color shades with blues positive and greys negative. Asterisks indicate the significance level *p*-value from Spearman’s *Rho* (*p* < 0.05 *, *p* < 0.01 **, *p* < 0.001 ***)*.* Environmental variables: depth of sampling (Z), water temperature (Temp.), salinity (Sal.), Chl *a* fluorescence (Fluo.), dissolved oxygen (O_2_), nitrate (NO_3_^2-^), silicate (SiO_4_), phosphate (PO_4_^3-^). Also given are values from the visual analysis of ice charts produced by the Canadian Ice Service (CIS): day-length (from Fig. 2); days between sampling (s) date and ice bridge collapsing (s-IBC); depth of maximum mean vertical gradient in fluorescence, dFluo./dz (ΔFluo.); pycnocline is the depth of Brunt-Väisälä frequency maximum (ΔN^2^); depth of the Surface Mixed Layer (SML); day of the year of when the polynya opened as open water (OW); day of the year when the samples were collected (s day); day of the year when new winter sea-ice (NWSI) covered the region; total days between OW and NWSI (OW-NWSI); days from the beginning of OW to sampling (OW-s).

**Supplementary Fig. S2.** **Microbial eukaryotic composition**. Relative abundance of the most abundant taxonomic groups from rDNA and rRNA in samples of all years (2005-2018) for each station and side. Sampling dates for the separate sides are arranged by month categories based on Fig. 3 db-RDA (main text) result. Surface communities are shown in the upper panels and subsurface chlorophyll maximum (SCM) communities in the lower panels.

**Supplementary Fig. S3. Samples clustered using Constrained Correspondence Analysis (CCA) with the Bray-Curtis dissimilarity measure**. Arrows indicate significant correlations between environmental parameters and community composition of samples: Temperature (Temp.), salinity (Sal.), dissolved oxygen concentration (O_2_), nitrate (NO_3_^2-^), silicate (SiO_4_), phosphate (PO_4_^3-^), depth of surface mixed layer (SML) and day-length. The rDNA and rRNA reads are analysed independently, with rDNA communities indicated by a black point in the center of the symbols; rRNA lack a central point; West (W°) Canadian side, East (E°) Greenland side. Month categories indicated by color.

**Supplementary Fig. S4. Community composition with samples clustered using Non-Metric multi-Dimensional Scaling (NMDS) with the Bray-Curtis dissimilarity measure**. The rDNA communities indicated by a black point in the center of the symbols; the rRNA points lack a central point; West (W°) Canadian side; East (E°) Greenland side. Year of sample indicated below the symbols, and month categories indicated by color.

**Supplementary Fig. S5. Pairwise CLAM test plot of OTUs comparing West versus East**. Canadian side samples (West, with n = 38) and Greenland side samples (East, with n = 45) of both depths: surface and subsurface chlorophyll maximum, all years (2005-2018) and from both rRNA and rDNA datasets. Note that OTUs with <10 reads were too rare to be categorised. Percentage is relative to total OTUs following the color code. The eight top OTU specialist by side are indicated as letters.

**Supplementary Fig. S6. Pairwise CLAM test** **plot of OTUs comparing surface versus SCM**. The test includes surface (n = 40) and subsurface chlorophyll maximum (SCM, n = 43) of both sides of the North Water, all years (2005-2018) and from both rRNA and rDNA datasets. Note that OTUs with <10 reads were too rare to be categorised. Percentage is relative to total OTUs following the color code. The three top OTU specialists by depth category are indicated as letters.

**Supplementary Fig. S7. Pairwise CLAM test plot of OTUs comparing rDNA versus rRNA**. The test includes rDNA samples (n = 44) and rRNA samples (n = 39) of both sides of the North Water, of both depths (surface and SCM) and all years (2005-2018). Note that OTUs with <10 reads were too rare to be categorised. Percentage is relative to total OTUs following the color code. The top OTU specialists by nucleic acid category are indicated as letters.

**Supplementary Fig. S8. Maximum Likelihood Dinoflagellata tree generated using RAxML based on partial length 18S rRNA gene sequences and reference long sequences**. The final RAxML evolutionary placement algorithm (EPA) placed short reads of target OTUs (red) into the reference tree. Taxonomic assignment was based on the nearest identified/annotated sequences (Supplementary Table S8). The reference tree was constructed using RAxML from an alignment of 94 sequences of 1,272 characters with bootstrap support repeated 1,000 times. Only bootstrap support >50 (of 100) is shown.

**Supplementary Fig. S9. A Maximum Likelihood *Micromonas* tree generated using RAxML *Micromonas* OTUs**. All 94 *Micromonas* 94 OTU sequences from this study were aligned with 4 reference of short length 18S rRNA gene sequences. Taxonomic assignment was based on the nearest identified/annotated sequences for the 4 reference sequences (Supplementary Table S8). The reference tree was constructed using RAxML from an alignment of the 98 sequences of 1,000 characters with bootstrap support repeated 1,000 times. Only bootstrap support >50 (of 100) is shown.

**Supplementary Fig. S10. Spearman correlations between eukaryotic communities of the 22 top OTUs (reads >0.5% of the entire community) from both rDNA and rRNA reads**. Spearman correlation coefficient values are represented by the size (large dots – high coefficients; small dots – low coefficients) and color (blue – positive; grey – negative) of dots. Number of asterisks represents the significance level *p*-value from Spearman’s *Rho* (*p* < 0.05 *, *p* < 0.01 **, *p* < 0.001 ***)*.*
